# Measurement of bound states in the continuum by a detector embedded in a photonic crystal

**DOI:** 10.1038/lsa.2016.147

**Published:** 2016-09-23

**Authors:** Roman Gansch, Stefan Kalchmair, Patrice Genevet, Tobias Zederbauer, Hermann Detz, Aaron M Andrews, Werner Schrenk, Federico Capasso, Marko Lončar, Gottfried Strasser

**Affiliations:** 1 Institute for Solid State Electronics, Vienna University of Technology, Floragasse 7, 1040 Vienna, Austria; 2 School of Engineering and Applied Science, Harvard University, Cambridge, MA, 02138, USA; 3 Centre de Recherche sur l’Hétéro-Epitaxie et ses Application, CNRS, Rue Bernard Gregory, Sophia-Antipolis, 06560 Valbonne, France

**Keywords:** embedded eigenvalues, photonic crystals, photodetector

## Abstract

We directly measure optical bound states in the continuum (BICs) by embedding a photodetector into a photonic crystal slab. The BICs observed in our experiment are the result of accidental phase matching between incident, reflected and in-plane waves at seemingly random wave vectors in the photonic band structure. Our measurements were confirmed through a rigorously coupled-wave analysis simulation in conjunction with temporal coupled mode theory. Polarization mixing between photonic crystal slab modes was observed and described using a plane wave expansion simulation. The ability to probe the field intensity inside the photonic crystal and thereby to directly measure BICs represents a milestone in the development of integrated opto-electronic devices based on BICs.

## Introduction

A two-dimensional periodic lattice of holes in a slab of material with a high refractive index, i.e., a photonic crystal slab (PCS), can exhibit a great number of fascinating optical phenomena^1, 2, 3, 4^. Some of these effects, which are intimately related to the in-plane periodicity of the photonic crystal, have been judiciously exploited to develop integrated photonic circuits^5^, enhance light-matter interactions^6^, create lasers with a lower threshold^7^ or superior far-fields^8^, design optical detectors with higher performance^9, 10, 11^ and even efficiently convert the frequency of light^12^. In the past, PCSs were often approximated as 2D photonic crystals in an effective index material, which is a useful model to describe the optical properties originating from the in-plane periodicity of a photonic crystal^13^. The problem with this model is that it completely ignores interference effects between the out-of-plane interfaces. Finite difference time domain (FDTD) simulations are able and frequently used to calculate the exact field distribution in PCSs but are time consuming and provide little insight. For these reasons, the existence of bound states in the radiation continuum (BICs) has been reported only recently, even though photonic crystal slabs have been subject to research for more than a decade. BICs have been demonstrated in photonic crystals first at the Γ-point, where the BIC originates from geometric symmetry^14^. Analogous phenomena, where a photonic bound state is decoupled from the continuum by symmetry, were reported earlier in other photonic structures, e.g., distributed feedback lasers and waveguide arrays^15, 16^. More recently, another type of BIC has been reported in PCSs, where the BICs are the result of accidental phase matching between in-plane and surface coupling waves at special points in the photonic band structure^17, 18, 19^. These off-Γ BICs exist at seemingly random wave vectors. Hence, the analysis of off-Γ BICs requires a model that includes not only geometric symmetry and in-plane coupling but also coupling via the continuum.

In this paper, we demonstrate direct measurements of BICs using a photodetector embedded into a PCS. We propose a 3D rigorous coupled wave analysis (RCWA)^20^ to analyze BICs and compare the theoretical results with the measured photonic band structure. This measurement technique allows us to directly measure the field intensity inside the PCS without an elaborate external optical setup. For the first time, this paper demonstrates an opto-electronic device that can directly measure optical bound states in the continuum. We discuss the fabrication of our PCS detector in the second section of this paper and explain the experimental setup. We compare the experimental measurements of the PCS band structures to those obtained using the 3D RCWA.

The photonic band structure of PCSs is far more complex than a 2D photonic crystal band structure. PCSs support higher order slab modes, which can couple to each other if they have some component of the same symmetry. To provide an intuitive understanding of the photonic band structure, we implemented a 2D plane wave model specifically designed for PCSs^21, 22^, which allows the analysis of mode symmetry and polarization mixing. In the third section of this paper, we describe polarization mixing in the photonic band structure. Finally, we report on the first direct measurement of a BIC using an embedded photodetector and provide a model for the detector response at the BIC based on temporal coupled mode theory.

## Materials and methods

Our PCS detectors are processed from an active photodetector material, consisting of a series of GaAs quantum wells separated by AlGaAs barriers. This type of photodetector is called a Quantum Well Infrared Photodetector (QWIP)^23, 24^. Band structure engineering allows precise control of the peak absorption wavelength and absorption magnitude. The GaAs/AlGaAs heterostructure of the QWIP is grown by molecular beam epitaxy on a semi-insulating GaAs substrate. First, a 2-μm-thick Al_0.85_Ga_0.15_As sacrificial layer is grown. Later, this layer is selectively etched to obtain a free standing slab that confines the modes vertically. The QWIP consists of 26 periods of 4.5-nm-thick GaAs quantum wells separated by 45-nm-thick Al_0.3_Ga_0.7_As barriers. The quantum wells are doped (σ=4 × 10^11^ cm^−2^) to supply carriers for the absorption process. Electrical contacts are formed by a 350-nm-bottom and 100-nm-top doped contact layer. The total thickness of the slab consisting of the quantum well heterostructure and both contact layers is 2 μm. This thickness is required to provide sufficient mechanical strength of the membrane. The design wavelength for the peak absorption of the QWIP is 8 μm.

**Figure 1:**
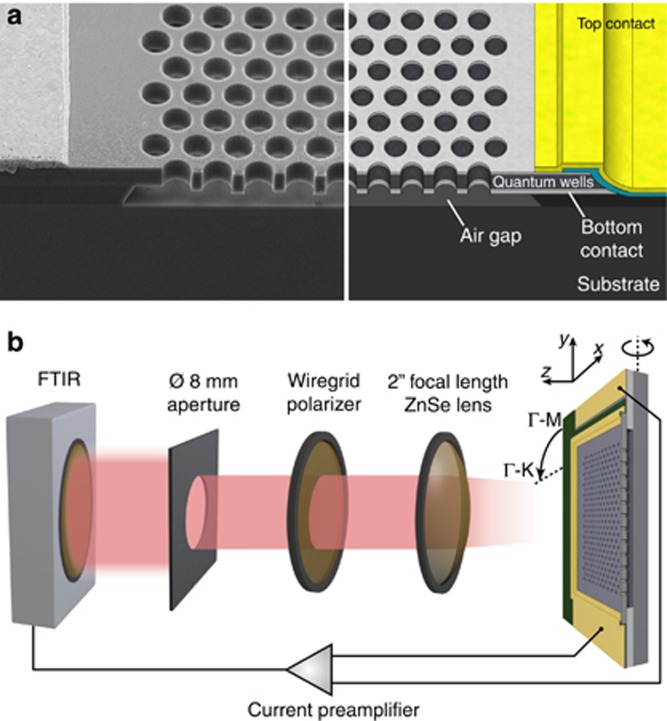
(**a**) SEM image of a cleaved PCS (left) and a schematic sketch of the device (right). The photonic crystal is processed into the active photodetector material. The photocurrent is collected via doped contact layers on the top and bottom of the suspended PCS membrane. (**b**) Measurement setup consisting of (left to right) the FTIR, an aperture, a polarizer, a lens and the measured PCS photodetector, mounted inside a liquid He cryostat.

Fabrication of the device starts with reactive ion etching of the photonic crystal holes through the slab into the sacrificial layer. The mesa for the device is chemically etched in a solution of H_3_PO_4_:H_2_O_2_:H_2_O=3:2:40. Top and bottom contact layers are electrically insulated with a 400-nm-thick Si_3_N_4_ layer deposited by plasma-enhanced chemical vapor deposition. To provide electrical contacts, windows are opened into the Si_3_N_4_ isolation by reactive ion etching and Ge/Au/Ni/Au metal contacts are deposited by electron beam evaporation. The sample is annealed at 430 °C to form ohmic contacts. The last processing step is selective under-etching of the slab with HCl (32%) to remove the sacrificial layer below the photonic crystal. Figure 1a shows a scanning electron microscope (SEM) image of a finished device (left) compared with a schematic sketch (right). Mechanical stress accumulated during the epitaxial growth limits the size of the photonic crystal to approximately 100 × 100 μm. Larger membranes tend to collapse during fabrication.

Figure 1b shows the measurement setup for the PCS photodetector. Spectral characterization of the device is performed using Fourier transform infrared spectroscopy (FTIR). Inside the FTIR, a Globar source emits broadband radiation in the mid-infrared. The light is focused onto the sample using a ZnSe lens with a 2” focal length. To narrow the incidence angle of illumination onto the sample, an 8 mm aperture is placed in front of the lens. The resulting opening angle is approximately 4°. The sample is mounted inside a liquid He cryostat to operate the photodetector at 30 K, which minimizes electrical noise through thermal carrier excitation^24^. After pre-amplification, the photocurrent from the sample is fed back into the FTIR to calculate the spectral response of the device.

The design of the device is similar to that of resonant photodetectors, but with a significantly higher doping concentration. A QWIP fabricated as a photonic crystal resonant photodetector would typically be doped two orders of magnitude lower^10^. In resonant photodetectors, the absorption coefficient is kept sufficiently low to achieve large resonant absorption enhancement and high performance gain^25^. In contrast, to accurately measure BICs, the doping concentration is tailored to be high enough that quantum well absorption is the dominating loss mechanism in the system. This point will be discussed in detail below. Because BICs arise from symmetry and phase matching, their existence is less dependent on in-plane losses.

The existence of BICs requires a structure with time-reversal symmetry ε(r)=ε*(r) and inversion symmetry *ε(r)=ε(-r)*^17^. We choose a photonic crystal slab with a hexagonal lattice, which fulfills these requirements^26^. The lattice constant *a* of the photonic crystal is chosen to be 3.2 μm, with the hole radius *r* being 0.2*a*.

**Figure 2:**
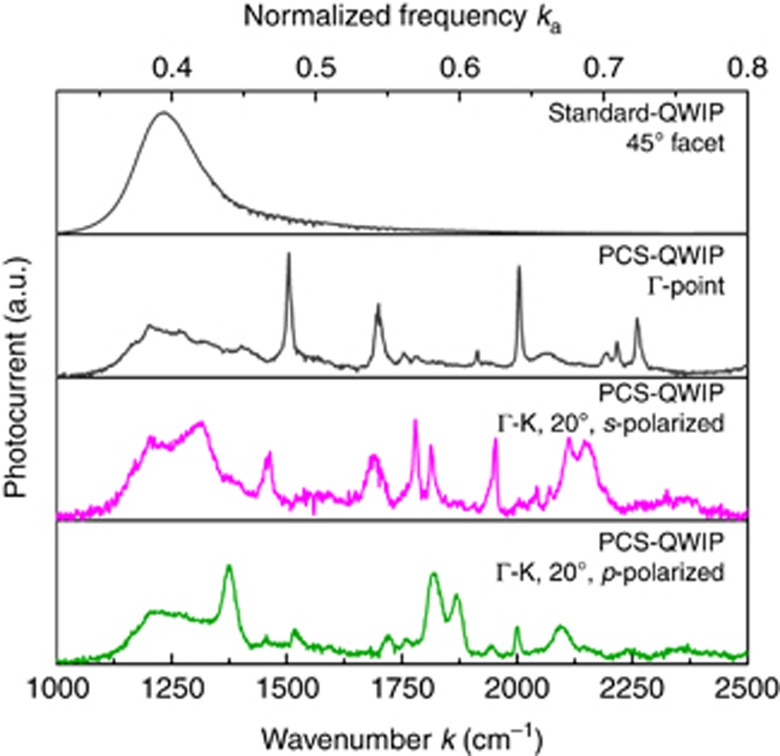
Normalized photocurrent spectra of a standard QWIP (top) and a PCS fabricated from QWIP material at surface normal incidence (2nd from top). PCS illuminated at 20° incidence along Γ-K for *s*-polarized (3rd from top) and *p*-polarized (4th from top) light.

The photonic band structure is measured by means of a band structure mapping^27, 28, 29^. Starting with the Γ-K direction, the device is rotated around the *y*-axis from 0° up to 70° in steps of 5°. At each step, a spectrum for *s*- and *p*-polarized light is measured. To also obtain the photonic band structure for Γ-M, the sample is then rotated by 90° around the *z*-axis. Figure 2 shows examples of measured spectra. For reference, a spectrum of a standard mesa QWIP device without a photonic crystal is included (Figure 2, 1st spectrum). In the spectra of the PCS device, several sharp peaks appear at the photonic crystal resonances (Figure 2, 2nd spectrum). For surface normal incidence at the Γ-point, the *s*- and *p*-polarized spectra are degenerate. For any other angle, the resonance peaks for *s*- and *p*-polarized light are non-degenerate (Figure 2, 3rd and 4th spectrum).

**Figure 3:**
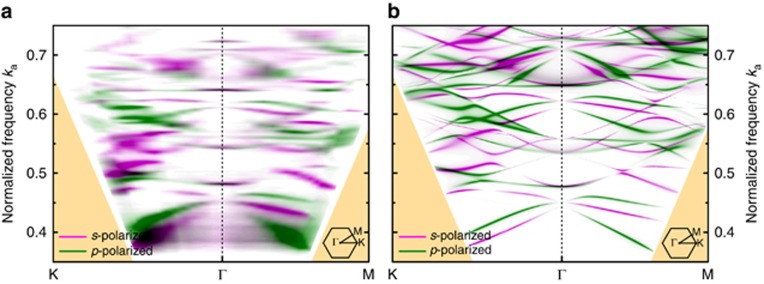
Photonic band structure of a PCS, measured as photocurrent from a PCS device (**a**) and simulated with RCWA (**b**). The yellow area indicates the light cone. Dimensions of the device: lattice constant *a*=3.2 μm, hole radius *r/a*=0.2 and slab thickness *d*=2 μm.

The measured spectra obtained from sample rotation are combined into a two-dimensional color plot, immediately showing the photonic band structure of the PCS (Figure 3a). For comparison, the band structure has been simulated via rigorous coupled wave analysis (RCWA) using the freely available S4 software^20^. The quantum well heterostructure is approximated as a homogenous and frequency independent medium, with a real part of the refractive index of *n*=3.12. Due to inter-sub-band selection rules, QWIPs absorb only photons polarized perpendicular to the quantum wells^30^. Therefore, the absorption was modeled with an anisotropic imaginary permittivity. To simplify the simulation, it was set as constant over frequency to *ε*_*i*_=0.05 in the *z*-direction, which corresponds to an average value for the bound-to-continuum transition at higher frequencies. The RCWA simulation results in a transmission and a reflection spectrum. The absorptivity of the PCS is calculated as 1-R-T. The simulated absorption spectra are combined into the photonic band structure in the same fashion as the measured spectra (Figure 3b). A comparison between the measured and simulated band structure shows excellent agreement between the fabricated device and the simulated structure.

In an ideal PCS, the quality factor of the resonance peaks can theoretically go to infinity. However, in real devices, the resonance width is limited by various losses. The total *Q*-factor of the resonator can be written as


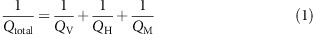


where *Q*_V_ is a measure of the coupling efficiency to incident light. In an ideal PCS without material absorption and an infinite lateral size, *Q*_V_ is equal to *Q*_total_. *Q*_H_ is the *Q*-factor derived from the horizontal in-plane losses due to the finite lateral extension of the PCS. *Q*_M_ represents the material losses, which in our case are dominated by the inter-sub-band absorption of the photodetector. The vertical *Q*-factor ranges from approximately 200 for resonances that couple efficiently to external fields and goes to infinity for resonances that do not couple at all. The horizontal *Q*-factor is limited by the finite size of the PCS. For the lateral device with dimensions of 100 × 100 μm, our PCS has 31 periods. From two-dimensional FDTD simulations, we determined that *Q*_H_ was well above 2000 for all resonances.

For light with constant intensity (i.e., no standing waves) in a homogenous material, the material quality factor is given by *Q*_M_*=Re*{*n*}/(2*Im*{*n*})^31^. The complex refractive index is related to the absorption by *α*=4*πIm*{*n*}/*λ*. At the photocurrent maximum of the QWIP, the peak absorption is approximately 1350 cm^−1^ for the provided doping^24^. At the absorption maximum at *λ*=8 μm, the material *Q*-factor is *Q*_M_ =20. The photonic crystal resonances are measured at higher frequencies, where the QWIP absorption is significantly lower. However, in the photonic crystal resonator, the electromagnetic field forms standing waves and the material is not homogenous. Furthermore, the absorption is non-zero only for an electric field in the growth direction. As a result, the effective absorption is given by


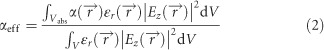


where *V*_abs_ is the volume of the absorbing material and *V* is the total volume^28^.

The in-plane photonic crystal mode profile and the vertical slab mode profile have great impact on the effective absorption. Modes that are localized mainly inside the holes have less overlap with the absorbing material. Modes, which are close to their respective cut-off frequency of the slab waveguide, exhibit large field components along the non-pure transverse-electric/transverse-magnetic (TE/TM) directions. For TM-like modes, the effective absorption is reduced by 50-70%. For TE-like modes, which only have a small electric field component in the *z*-direction, the effective absorption is reduced to less than 1%. Owing to the small effective absorption for TE-like modes, the measured photonic band structure shows mainly TM-like modes. This effect helps greatly to identify the measured resonances when comparing to simulations.

Near the absorption maximum of the photodetector, the measured *Q*-factor is dominated by the material *Q*-factor. At higher frequencies, the material absorption is smaller and all *Q*-factors are in the same order of magnitude. Therefore, the measured *Q*-factor near the absorption maximum is approximately 100 and goes up to 500 at higher frequencies.

## Results and discussion

The photonic band structure of PCSs is complex compared with a purely 2D photonic crystal. Accidental phase matching between out-of-plane and in-plane waves gives rise to BICs. PCSs support higher order slab modes, which can couple to each other. This leads to polarization mixing and causes distortions of the photonic bands. Especially at frequencies at which modes from multiple slab orders overlap, the band structure is strongly distorted by polarization mixing and difficult to understand. To provide an intuitive understanding of the photonic band structure, we have computed the PCS photonic band structure using the revised plane wave expansion method (RPWEM)^21, 22^. This method accounts for the slab wave guiding by using an effective refractive index^13^. However, it does not include 3D effects such as BICs and polarization mixing. Comparison of the RCWA and RPWEM simulated band structures allows the identification of BICs and polarization mixing throughout the entire photonic band structure.

**Figure 4:**
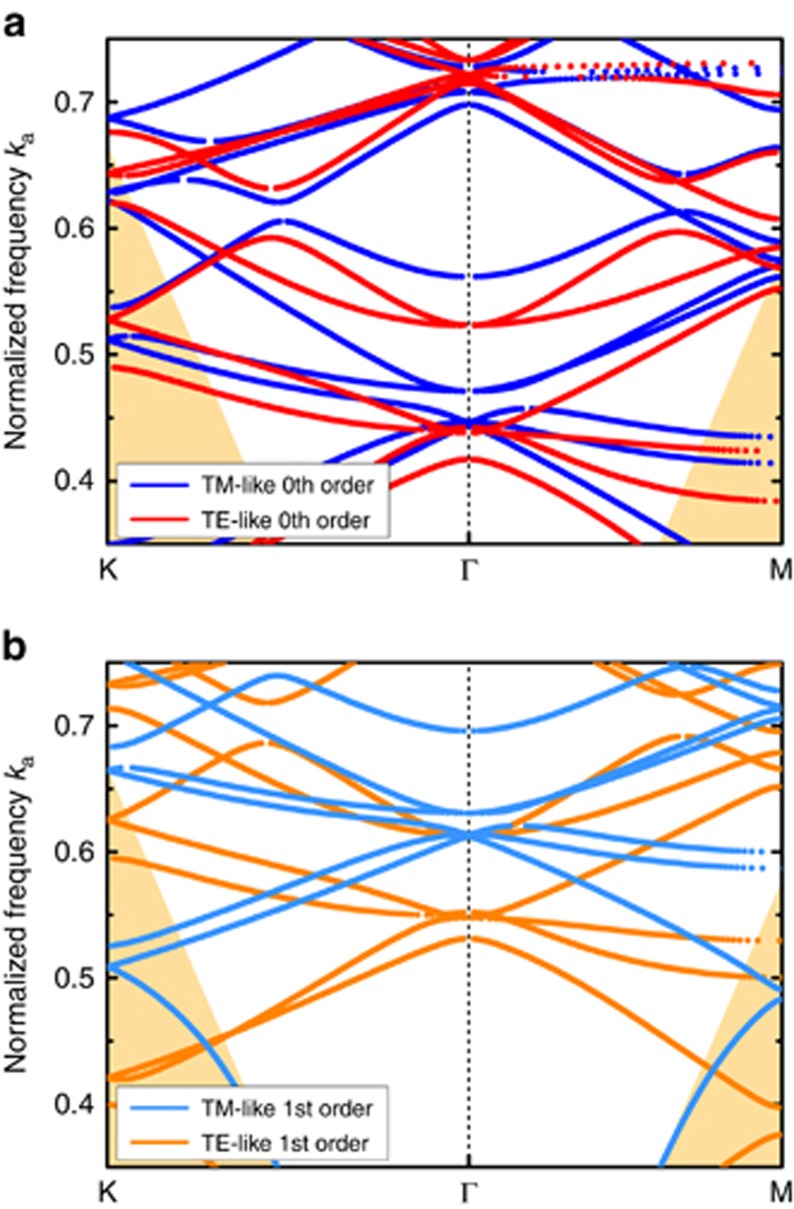
Simulated photonic band structure for the 0th order (**a**) and 1st order (**b**) slab mode. The photonic bands of the 1st order slab mode are blue-shifted to higher frequencies with respect to the 0th order band structure.

The photonic band structure depends on the vertical mode profile in the slab. A separate photonic band structure exists for each slab mode. The confinement in the *z*-direction induces a blue-shift of the photonic crystal modes. Compared with the fundamental mode, the confinement of higher order modes is weaker and the blue-shift is stronger^32^. In our structure, only the fundamental 0th order modes and the 1st order modes overlap in frequency with our photodetector absorption. Figure 4 compares the TE- and TM-like bands for the 0th order and 1st order slab mode. The photonic bands of the 1st order slab mode are blue-shifted to higher frequencies with respect to the 0th order band structure.

Interactions between bands from different slab modes result in the formation of anti-crossings^27^. Owing to the large number of additional bands originating from higher order slab modes and subsequent polarization mixing, the real photonic band structure is strongly distorted when compared with a 2D photonic crystal band structure without vertical confinement. The out-of-plane symmetry (along the *z*-axis) of the electromagnetic field in the PCS determines if polarization mixing occurs. Modes with even symmetry do not interact with odd symmetry modes^33^. Therefore, the symmetric TM-like slab modes do not interact with the anti-symmetric TM modes. The same argument applies for the TE-like modes. However, symmetric TM-like modes have the same vertical symmetry as anti-symmetric TE-like modes, and polarization mixing between these modes is possible.

**Figure 5:**
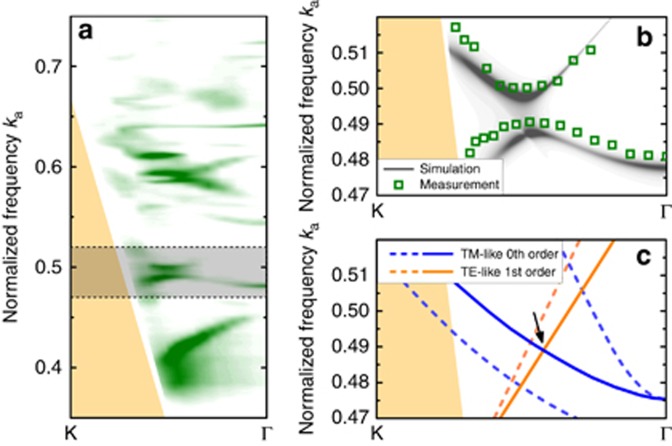
(**a**) Photonic band structure between K and Γ for *p*-polarized light. The shaded area indicates polarization mixing between two photonic bands. (**b**) The resonance peaks (green squares) of the measured photonic band structure are in good agreement with the RCWA simulation (gray). (**c**) Comparison with the RPWEM simulated photonic band structure allows for identification of the anti-crossing (arrow) as an interaction between a 0th order TM-like band (solid blue line) and a 1st order TE-like band (solid orange line). The dashed lines indicate modes that do not appear in the measured photonic band structure because they couple only weakly to external fields.

In Figure 5a, an anti-crossing in the *p*-polarized measured band structure is indicated by a shaded box. Figure 5b shows a close-up view of the shaded area. The gray lines are the results of the RCWA simulation and the green squares indicate the position of the measured resonance peaks. Comparison with the RPWEM simulation (Figure 5c) identifies the anti-crossing as an interaction between a 0th order TM-like mode (solid blue line) and a 1st order TE-like mode (solid orange line).

BICs were first observed in electronic superlattices^34^ and recently also in photonic crystals^17, 18, 19, 35^. BICs in photonic crystals are the result of destructive interference between photonic crystal modes. The PCS modes can be coupled through in-plane wave propagation (non-radiative modes) or via the continuum within near fields (radiative modes). The BIC is formed when the radiation from all possible channels interferes destructively, causing the overall radiation to vanish.

**Figure 6:**
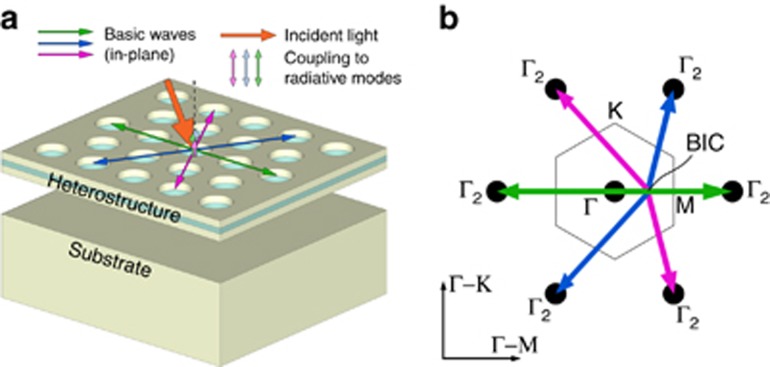
Formation of BICs in photonic crystals. (**a**) PCS modes can be decomposed into basic waves. The basic waves are coupled to each other through in-plane wave propagation or via the continuum within near fields. The BIC is formed when all possible channels interfere destructively. (**b**) Basic waves in k-space. In the Γ-K direction, all radiative contributions cancel out because of symmetry. In the Γ-M direction, the symmetry is broken. These components can cancel as a result of accidental phase matching at special points in the photonic band structure, leading to the formation of BICs. The gray hexagon in the center indicates the Brillouin zone of the hexagonal lattice.

BICs can be modeled using coupled wave theory, as described by Yang et al.^19^. To understand the formation of BICs, we need to identify the dominant radiation channels. From Bloch’s theorem, we know that all PCS modes are Bloch wave states. We can express the Bloch wave state as a Fourier series of plane waves, which are referred to as basic waves^36^. Figure 6a illustrates the propagation of basic waves in the PCS and the coupling to the incident light via radiative modes.

We can develop an intuitive picture of BIC formation by considering only the basic waves close to the second-order Γ point, as shown in Figure 6b. We assume that the radiation from these Γ_2_ waves is the dominant contribution to the total radiation^37^. This approximation works best for structures with low index contrast. In the Γ-K direction, all radiative contributions cancel out because of symmetry. This is not the case for the Γ-M direction. These components cancel out through destructive interference of basic waves via coupling to radiative modes, which happens as a result of accidental phase matching^19^. These conditions can be fulfilled at many points in the photonic band structure.

Previous measurements of BICs were dependent on reflection measurements. In our device, we can directly measure the intensity inside the photonic crystal using the embedded photodetector. Because *Q*_V_ becomes very large at these points we cannot simply extract it from the measured resonance width. When the wavelength of the incident light is close to that of BICs, *Q*_total_ is always dominated by *Q*_M_, i.e., the absorption of the photodetector. Instead, we measure the change in photocurrent, which is directly related to *Q*_V_ because of the decrease in in-coupling efficiency.

**Figure 7:**
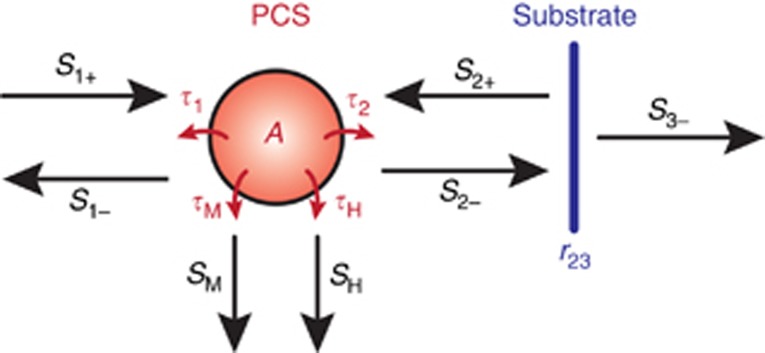
TCMT model of the PCS photodetector. *A* is the resonance of the PCS. The substrate is represented by the reflection coefficient *r*_23_. Backward and forward running waves are coupled to the resonance by the respective lifetimes *τ*. Material absorption and in-plane loss are included through the loss channels *S*_M_ and *S*_H_.

The relation between absorption and the vertical *Q*-factor can be deduced using temporal coupled mode theory (TCMT)^38^. Figure 7 shows a simplified model, in which the PCS is modeled as a resonance *A* with lifetimes *τ*_1_ and *τ*_2_ for coupling from top and bottom. Together, these two lifetimes compose the vertical lifetime *τ*_1_=*τ*_2_=2*τ*_V_. The *Q*-factor is related to *τ* by *Q*=*ωτ*/2.

The material absorption and in-plane loss channels are added to the resonance through the lifetimes *τ*_M_ and *τ*_H_, respectively. The substrate at the distance *h*_2_ is represented by a reflection coefficient *r*_23_. Light in the air gap between the PCS and substrate propagates with the propagation constant *β*_2_. *r*_slab_ and *t*_slab_ are the complex reflection and transmission coefficients of the slab, respectively. The absorption is modeled as the transmission from the incoming light channel *S*_1+_ to the material loss channel *S*_M_. For the photodetector, this corresponds to the absorption quantum efficiency *η*:


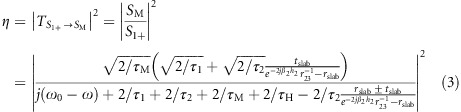


To study changes in the vertical *Q*-factor in the presence of an absorber at the BIC, we consider the case *Q*_V_>>*Q*_M_, which is equivalent to *τ*_1,2_>>*τ*_M_. In this limit, the dominating term in the denominator of Equation (3) is 2/*τ*_M_. For constant absorption, the photocurrent amplitude is then determined by the terms 2/*τ*_1_ and 2/*τ*_2_ in the numerator. With the photoconductive gain *g*_*ph*_, the measured photocurrent is related to the incident photon flux *ϕ* by^24^


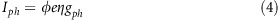


and from this, we obtain


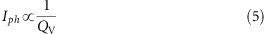


Hence, the photocurrent is inversely proportional to the vertical *Q*-factor.

**Figure 8:**
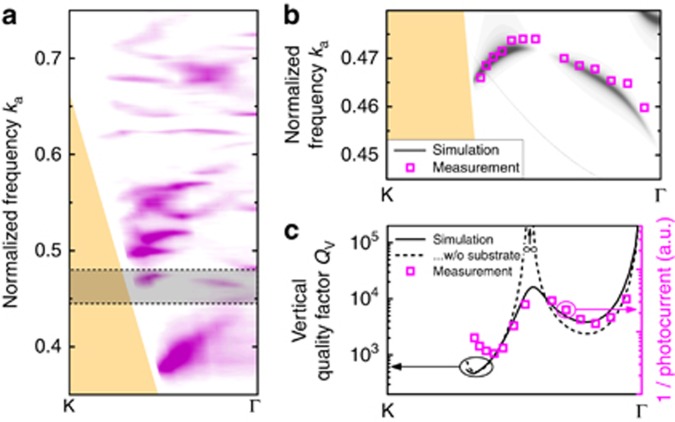
(**a**) Measured photonic band structure between K and Γ for *s*-polarized light. The gray area indicates a BIC. The photocurrent vanishes when moving along the photonic band from K to Γ and reappears after passing the BIC. (**b**) Magnified view of the gray area in panel **a**. The resonance peaks (pink squares) of the measured photonic band structure are in excellent agreement with the RCWA simulation (gray). (**c**) At the BIC, the photocurrent vanishes. Without substrate (dashed gray line), the vertical *Q*-factor becomes infinite at this point. With substrate (solid line), the vertical symmetry is broken and the vertical *Q*-factor remains finite.

The measured photonic band structure shows several occurrences of BICs where the photocurrent goes to zero when moving along the band. Figure 8a shows the measured photonic band structure in the Γ-K direction for *s*-polarized light. The shaded area denotes a photonic band with a BIC. This photonic band is relatively flat, which means that the absorption of the photodetector and therefore the material *Q*-factor *Q*_M_ remains constant over the entire range of *k*-vectors.

The peaks of the measured photonic band overlap well with the simulated photonic band structure (Figure 8b). At the center of the photonic band, the photocurrent vanishes, indicating a BIC. The inverse of the measured photocurrent 1/*I*_*ph*_ is in excellent agreement with the vertical *Q*-factor *Q*_V_ predicted by the TCMT (Figure 8c). Because the substrate in our device breaks the vertical symmetry of the structure, the vertical *Q*-factor remains finite (solid line), as opposed to a perfectly symmetric structure (dashed line)^35^. However, *Q*_V_ still exceeds 10^4^, which is sufficiently high to observe a vanishing photocurrent at the BIC. The deviation of the measured photocurrent from the simulation at high measurement angles near the light cone is caused by the cryostat window, which cuts off a fraction of the incident light.

## Conclusions

We designed and fabricated a photonic crystal slab with an embedded QWIP photodetector. This technique allows us to directly measure the light intensity inside the structure and calculate the photonic band structure from measured photocurrent spectra. Comparison of the RCWA and RPWEM provided us with an intuitive understanding of the photonic band structure. Our RCWA simulation accurately describes BICs and polarization mixing. We reported the first direct measurement of a BIC in an opto-electronic device and provided a model for the detector response at the BIC based on temporal coupled mode theory.

In addition to the fundamental aspect of the effects described in this paper, the ability to directly measure the field intensity inside a PCS represents an important milestone in the development of integrated opto-electronic devices using BICs, particularly for compact resonant sensing devices, in which a resonator is used to enhance the sensitivity. Such systems are found, e.g., in laser spectroscopy and environmental monitoring^39^. These systems depend on low intrinsic absorption losses, which makes it unfeasible to embed the detector directly into the resonator. Sensors based on BIC photodetectors could open up a new class of compact sensing devices. Such devices would benefit from the extremely high *Q*-factor at the BIC, which is a result of phase matching and symmetry. Anything breaking the symmetry would be registered as a huge signal increase by the photodetector. Such a device could be used as a highly sensitive integrated particle detector for environmental sensing.

Future experiments could explore the effects of BICs coupled with photonic crystal defects such as waveguides and cavities. With the ability to measure the field intensity in a photonic crystal slab, our device is well suited to study these systems. Future research could also focus on lasers, nonlinear optics and optics-mechanics based on BICs.
